# On Chip Optical Modulator using Epsilon-Near-Zero Hybrid Plasmonic Platform

**DOI:** 10.1038/s41598-019-42675-z

**Published:** 2019-04-30

**Authors:** Mohamed A. Swillam, Aya O. Zaki, Khaled Kirah, Lamees A. Shahada

**Affiliations:** 1 Department of Physics, School of Science and Engineering, The American University in Cairo, New Cairo, 11835 Egypt; 2 Engineering Physics Dept., Faculty of Engineering, Ain Shams University, Abassia, Cairo 11517 Egypt; 3 Department of Chemistry and Earth Sciences, College of Arts and Science, Qatar University, P.O. Box, 2713 Doha, Qatar

**Keywords:** Microresonators, Silicon photonics

## Abstract

In this work, we propose a micro-scale modulator architecture with compact size, low insertion loss, high extinction ratio, and low energy/bit while being compatible with the silicon-on-insulator (SOI) platform. This is achieved through the utilization of epsilon-near-zero (ENZ) effect of indium-tin-oxide (ITO) to maximize the attainable change in the effective index of the optical mode. It also exploits the ITO layer in a hybrid plasmonic ring resonator which further intensifies the effect of the changes in both the real and imaginary parts of the effective index. By electrically inducing carriers in the indium tin oxide (ITO), to reach the ENZ state, the resonance condition shifts, and the losses of the hybrid plasmonic ring resonator increases significantly. This mechanism is optimized to maximize the extinction ratio and minimize the insertion loss. The proposed structure is designed to maximize the coupling to and from standard SOI waveguide, used as access ports. In addition, the operational region is reconfigurable by changing the bias voltage.

## Introduction

Over the past decade, research has focused on fabrication of photonic integrated circuits (PICs) on silicon (Si) substrates for data transmission applications such as, long-haul telecommunications^1,2^, short-reach data communications^3,4^ and interconnects^5,6^ for inter/intra-chip optical communication. Silicon photonics is considered as a suitable photonic platform for large scale photonic integrated circuits (PICs) such as optical interconnects, optical switches, and wavelength De/multiplexers. This is mainly due to the ability to fabricate low loss structure with minimum surface roughness and high yield. This was made possible due to the utilization of the mature, and well-developed complementary metal-oxide-semiconductor (CMOS) fabrication and packaging processes typically used in very large scale integrated electronic circuits. However, the weak non-linear effects of silicon as an electro-optical material is one of the most important drawbacks of this platform. Due to the weak electro-optic (EO) effect of silicon, silicon electro-optical modulators (EOM) suffer from large footprints, hence, high driving capacitance. The speed of the modulator and its power consumption require the minimization of the driving capacitance. Microring resonance-based modulators have shown great potential to reduce the footprint of Si modulators because the light passes multiple times in the waveguide allowing enhanced interaction compared to single-pass devices such as Mach-Zehnder Interferometer (MZI) modulator^7–10^. Recently demonstrated silicon microring modulators have footprints as low as 500 µm^2^ and power consumption less than 10 fJ/bit^11–13^.

In principle, EO modulation of the ring resonators can be achieved by the variation of the effective index of the ring waveguide which varies the resonance condition of the ring^11–16^, It is desirable to have high quality factor rings to minimize the energy per bit needed to achieve certain extinction ratio (ER) which increases the modulation efficiency (ER/V). However, on one hand, high quality factor resonators are sensitive to temperature and process variations and have limited optical bandwidth. Thus, typical index modulated ring modulators exhibit an inherent trade-off between their optical bandwidth and the modulation efficiency.

On the other hand, electro-absorption (EA) modulators can have wider optical bandwidth and controlled ER. Indium-tin-oxide (ITO) has been recently utilized as the absorbing media in such modulators, due to its fast carrier dynamics, and large number of accumulated carriers which results in high absorption under bias. This is also combined with large change in the refractive index. The ITO can be electrically tuned to reach ENZ state at the near infrared range^17^. Recent work shows that the optical dielectric constant of ITO in the charged layer of a metal-oxide-conducting oxide (MOS) structure can be electrically controlled over wide wavelength^17–21^. Once an electric field is applied to the MOS structure, the induced surface charge can greatly change the optical properties at the interface between the oxide and the ITO. The formation of the electron accumulation layer at the interface between the dielectric and the conducting oxide is a fast process. Given the small thickness of the oxide in a MOS configuration, the drift velocity is usually high enough to ensure the accumulation layer formation speed is well above 1 THz. Thus, electro-optical modulators that utilize conducting oxides are capable of supporting speeds in the THz regime^20,21^. For this EA based modulator, the ER and the modulation efficiency increase with increasing the length of the modulator^17–21^. This in turn increases the capacitance of the modulators and limits its modulation speed.

Thus, our main goal is to provide a design that mitigate the need for high quality factor resonators so that we can achieve high efficiency modulation with moderate or low quality factor cavities. The main challenge is the possibility to achieve these requirements with low energy/bit and high modulation speed.

**Figure 1:**
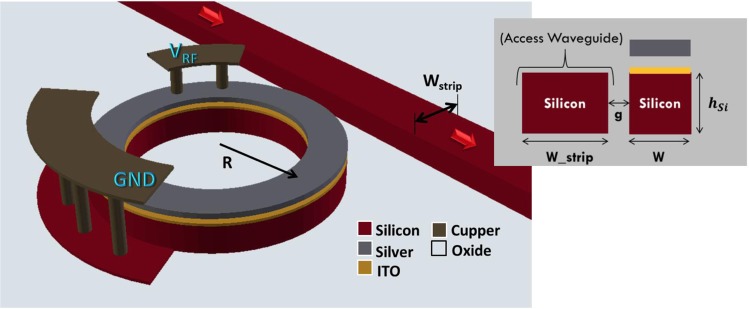
Three-dimensional structure of the modulator device, (inset) 2D sketch of the coupling region showing the cross section of the ring hybrid plasmonic waveguide (W) and pure silicon access waveguide (W_strip) separated by a gap (g).

This was made possible through our proposed design, shown in Fig. 1, that exploits both index change and loss effect in a critically coupled hybrid plasmonic ring resonator based modulator. The ITO is exploited to introduce the large index and loss change in the ring. The ring is designed to achieve critical coupling resonance for maximum ER performance. The coupling to and from the ring is made through a standard SOI waveguide for better integration with SOI platforms. The insertion loss, and coupling is optimized to reach high ER, low insertion loss, and high modulation speed with low energy consumption.

## Operation Theory

Controlling the internal round trip loss of a ring resonator around the critical coupling has been recently proposed in^22^. The power transmitted through an access waveguide connected to the ring resonator in a single bus configuration is described by:

1 $${P}_{out}={P}_{in}\frac{{\alpha }^{2}-\alpha t\,\cos \,\varphi +{t}^{2}}{1-2\,\alpha \,t\,\cos \,\varphi +{\alpha }^{2}{t}^{2}}$$

where *P*_*in*_ is the input optical power, *α* is the round-trip transmission coefficient, *t* is the transmission coefficient of the ring-waveguide coupler and *ϕ* is the round-trip phase shift.

The transmitted transfer function at the resonance wavelength (*ϕ* = *Zero*) is expressed by:

2 $$T({\lambda }_{res})={(\frac{t-\alpha }{1-\alpha .t})}^{2}$$

**Figure 2:**
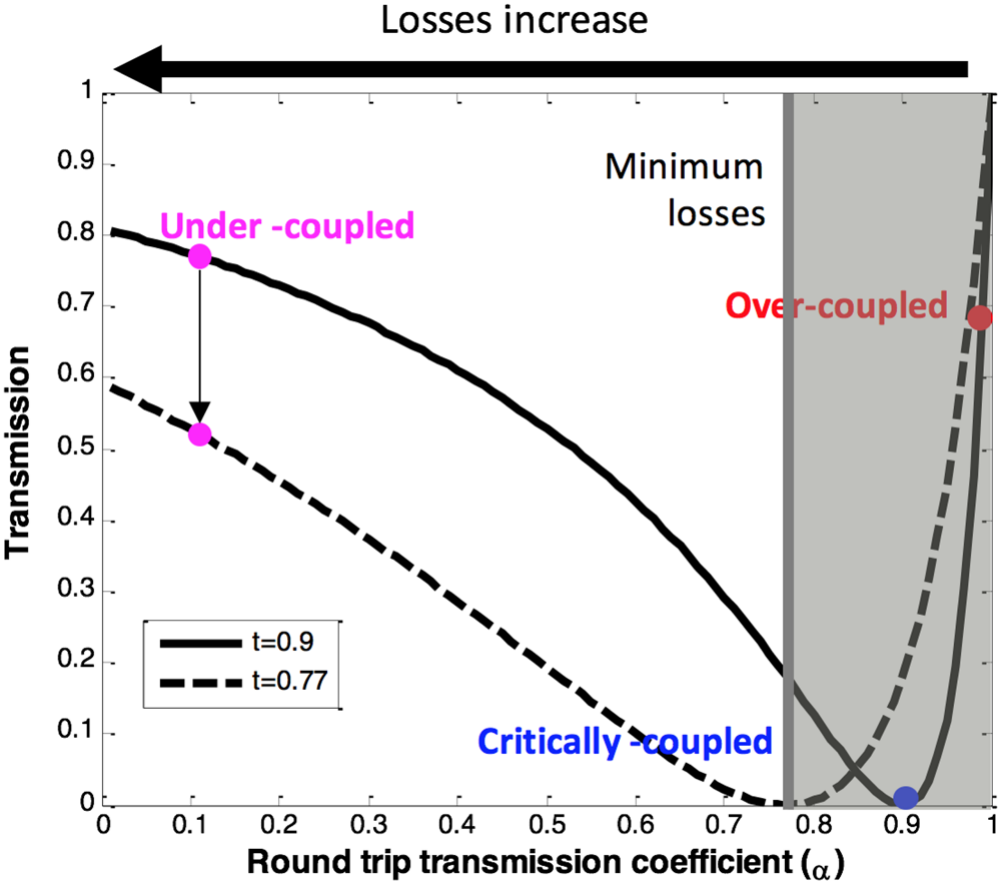
On-resonance transmission versus the round trip transmission coefficient. White area showing the allowed operation area for low-Q ring modulator.

Figure 2 shows the on-resonance transmission versus the round trip transmission coefficient (*α*) for (*t* = 0.9). Our goal is to achieve critical coupling condition at which the transmitted power at the resonance wavelength is zero to maximize the ER of the proposed modulator. A critically coupled resonator loses the same power to the intrinsic loss mechanisms as to the coupling to the external access waveguide i.e. t is equal to α^22^.

To investigate the design trade-offs in a loss-modulated ring resonator. The on-resonance transmission characteristics are depicted for different coupling coefficients in Fig. 2. It is worth mentioning that lower round-trip losses offer devices with steeper transmission characteristics (better voltage sensitivity) as well as lower insertion losses.

Modulation of the round trip loss^22^ or the coupling coefficient^23^ previously demonstrated took advantage of the transmission curve steepness in low-loss resonators where the device operation was switched between the over-coupled and critically coupled conditions. On the other hand, few research work has focused on the loss modulation of resonators of low quality factor since they suffer from a trade-off between the insertion loss and the modulation efficiency^24^.

In our proposed design, we consider a hybrid plasmonic (HP) waveguide ring modulator. This device utilizes a conducting oxide as the electro-optical material of which the real and imaginary parts of the refractive index change significantly in response to applied voltage^23^. The proposed device is enhanced by trapping the light in the formed accumulation layer by utilizing a hybrid plasmonic waveguide which significantly boosts the light matter interaction. Using both index modulation and loss modulation, the proposed electro-optical modulator provides a good compromise between speed, insertion loss and modulation efficiency.

To choose the optimum coupling condition for the modulator, we determine the maximum possible round-trip transmission. Then, choose the coupling coefficient t that gives the best performance for the given round-trip losses. The transmission sensitivity $\partial {{\rm{T}}}_{{\rm{dB}}}/\partial {{\rm{\alpha }}}_{{\rm{dB}}}$ as a function of t is given as:

3 $$\frac{\partial {T}_{dB}}{\partial {\alpha }_{dB}}=\alpha \times \frac{{t}^{2}-1}{(t-\alpha )(1-\alpha t)}$$

The modulator device efficiency depends on the voltage sensitivity. We can define the voltage sensitivity as:

4 $$\frac{\partial T}{\partial V}=\frac{\partial T}{\partial \alpha }\times \frac{\partial \alpha }{\partial V}$$

As can be shown from (3), the operation near the critical coupling offers the highest voltage sensitivity to any small signal changes in the round trip losses.

## Device Structure

The proposed modulator is based on a hybrid plasmonic ring resonator with a thin ITO layer over the silicon waveguide as shown in Figure 1. The thickness of the ITO layer is 10 nm. An oxide layer of 20 nm thickness lies between the ITO and the top silver forming a MOS (metal-oxide-semiconductor) structure. The light is coupled to this ring resonator via a conventional silicon waveguide of height 220 nm which is standard in the silicon photonic platform. The modulator has two electrical electrodes; the RF signal is connected to the silver and the silicon is connected to the ground. Connections to the probes are done using conventional electrical copper via.

## Modal Analysis and Loss Calculations

**Figure 3:**
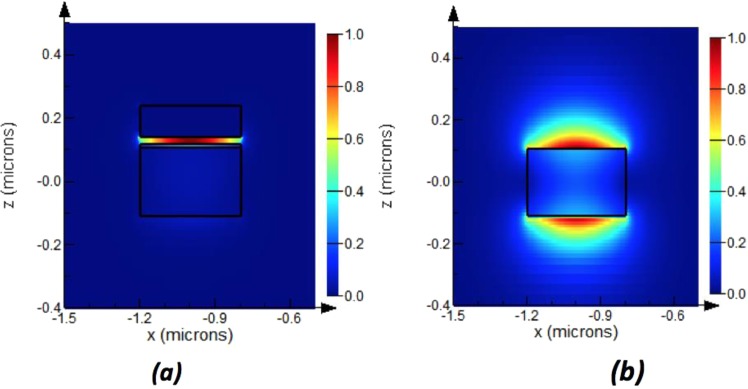
Electric field intensity for width = 400 nm, (**a**) Hybrid plasmonic waveguide 1st mode, (**b**) TM mode of a silicon waveguide.

Using commercial tool, Lumerical, for solving Maxwell’s equations using finite difference method to calculate the eigenmodes of the waveguides^25^, the modes of the hybrid plasmonic waveguide (HPW) are systematically studied. The electric field is confined in the upper oxide layer as shown in Fig. 3-a. The effective index of the hybrid plasmonic mode increases as the waveguide width (W) increases. The modal losses of the fundamental order TM HPWG mode are estimated by the mode solver in the range of 0.1 to 0.12 dB/µm. The low loss of HPW is expected and known feature for such waveguides^18,20^. A second order TM hybrid plasmonic mode exists once the width reaches 400 nm.

Since the light is coupled through a conventional silicon waveguide, the effect of waveguide width on the effective index of its TM and HPWG modes is studied at the operational wavelength of 1550 nm. The coupling between the hybrid plasmonic mode and the access silicon waveguide is maximized through phase matching of the two waveguides.

To investigate the potential of using the HP waveguide in ring resonators, we need to calculate its bending losses. The bending losses mainly consists of radiation loss and absorption loss. The total losses of the bend determine the intrinsic quality factor (Q_intrinsic)_ of the ring resonator which can be calculated as

5 $${Q}_{int}=\frac{2\pi {n}_{g}}{\lambda .{\alpha }_{av}}$$

where N_g_ is the group index, λ is the operating wavelength of 1550 nm and α_av_ is the imaginary part of the propagation constant corresponding to the average loss.

**Figure 4:**
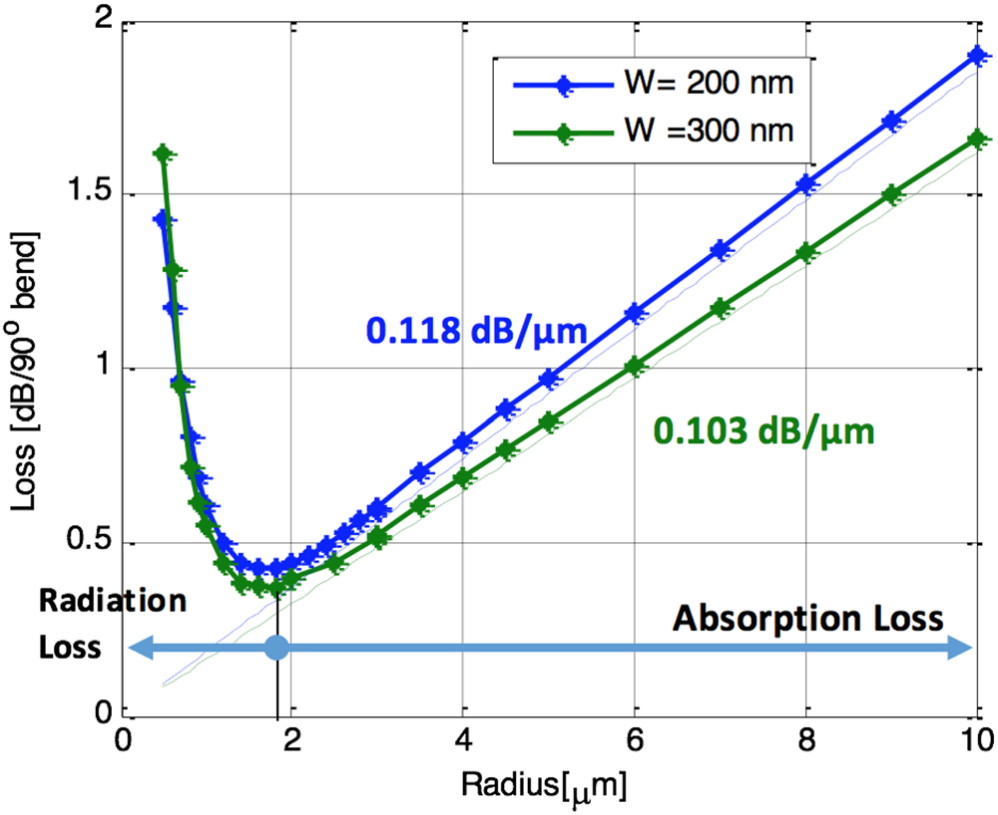
Bend losses versus the bend radius for the ITO hybrid plasmonic waveguide.

**Figure 5:**
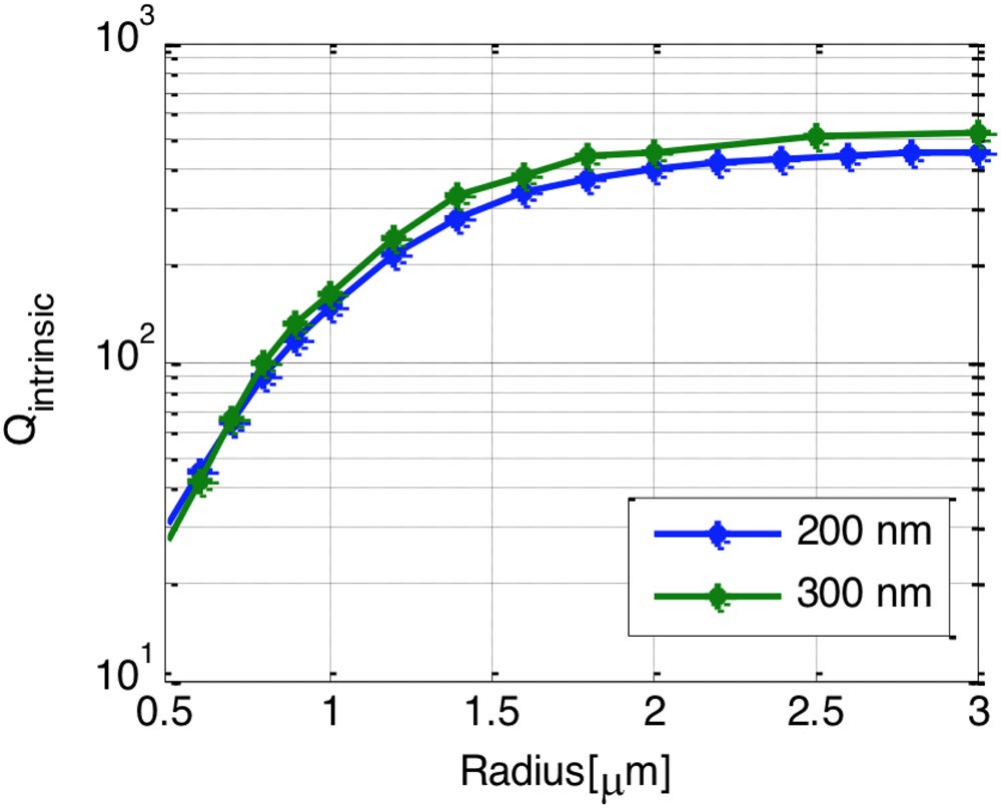
Quality factor versus the bend radius for the ITO hybrid plasmonic waveguide.

Figure 4 show the relationship between the radius and the total loss in a 90° bend calculated by 3D electromagnetic full wave simulation using Finite Difference Time Domain^26^. Here, we considered HP waveguides of widths 200 nm and 300 nm. The thickness of the ITO layer is taken to be 10 nm, the oxide thinness is taken to be 20 nm. The thinness of the silicon layer, *h*_*Si*_, is taken to be 220 nm. For the FDTD simulations, a numerical convergence study has bene performed to insure that the effect of the mesh size has no effect on the results. This study is exploited to determine the maximum mesh size that maintain the accuracy of the results within 5% maximum change. Accordingly, the mesh size in the vertical direction, Z is taken to be 2 nm. While in the mesh size in the lateral direction, X, and the propagation direction, Y, is taken to be 10 nm. A perfect matched layer with 8 layers in each side is utilized as a boundary condition. Using these simulations, we may conclude that the loss decreases as the radius increases until the losses reach a minimum and then starts to increase again. The permittivity for the ITO is used as described in the method section hereafter. If the radius is large enough the losses can be identical to the propagation losses of the straight waveguide mode (dotted lines). Hence, the bend losses are dominant in the small radii region and the propagation losses are dominant in the large radii region. The corresponding intrinsic quality factor variation with the bend radius is plotted in Fig. 5.

For our modulator application, we choose the highest quality factor that doesn’t limit the speed of the modulator. For an estimated capacitance per unit area of 0.93 fF/µm^2^ and the fast formation of the accumulation layer, the speed can reach 0.5–1 THz. Increasing the radius of the ring increase the total capacitance of the modulator and limit the modulation speed. Hence a radius of 1 um and a waveguide width of 200 nm is chosen to minimize the capacitance and keep low loss of the system. These designs parameters are corresponding to an intrinsic quality factor ~200 and total quality factor of the order of <100. In this case the optical bandwidth is around 20 nm which is wide enough to allow high speed modulation.

## Electrical Modulation

**Figure 6:**
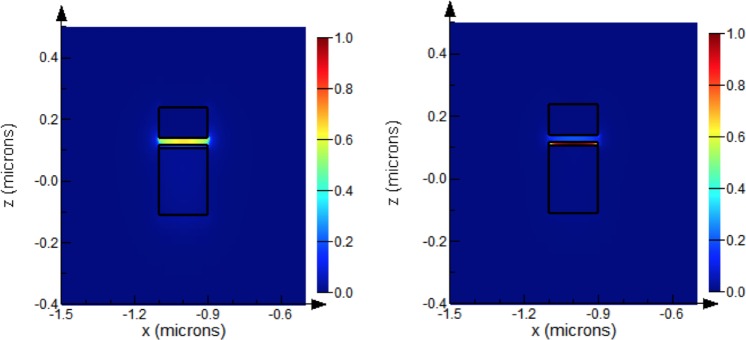
Electric field intensity profile of the 1st order TM Hybrid plasmonic mode for width 200 nm when a) V = 0 V, b) V = 2 V.

Upon applying an electrical potential across the ring MOS structure, an accumulation layer is formed at the interface between the ITO and the oxide. To estimate the permittivity of the ITO under applied voltage a quantum treatment self-consistently solves Poisson’s equation (for potential) and Schrodinger’s equation for bound state energies and carrier wave functions has been utilized as described in^27^. Using this model, the permittivity of the ITO can be calculated for any level of biasing. For example, these values are calculated in Table 1 for of 2 V and agree with those in^18,23^. Hence, the effective index and the propagation losses can be estimated using finite difference mode solver^25^ at any applied voltage level. For this solver, the same mesh size and boundary condition described above for the FDTD has been utilized to insure consistency. As clearly indicated in^18,23,27^, once an external voltage is applied on this configuration, the ITO starts to accumulate free carriers and moves from a dielectric state to a metal like state where the real part of the refractive index drops and the imaginary part increases. Under such condition, around the wavelength of 1550 nm, the real part of the permittivity approaches zero (ENZ) and the imaginary part increase dramatically. Hence, the electromagnetic field tends to be confined more in the ITO/ oxide interface with more penetration in the thin ITO due to its lower refractive index compared to the oxide as shown in Fig. 6. By working in the ENZ region, the effect of the loss on the electromagnetic field is maximized as required. Table 1 shows the change in the carrier concentration, the plasma frequency of the ITO, the change in the effective index and losses of the HPW mode when the width is 200 nm.

**Table 1:** Comparison of the On and Off states of the modulator.

	*N*_*c*_ [*cm*^−3^]	*ω*_*p*_ [red/sec]	*n*_*eff*_	Loss [dB/µm]
V = 0 V	1 × 10^19^	3 × 10^14^	2.14 + j 0.0036	0.128
V = 2 V	0.68 × 10^21^	2.47 × 10^15^	2 + j 0.36	2.7

The mode propagation losses increase dramatically to ~20 times the off state. This is due to the electric field becoming more confined in the accumulation layer when the voltage is applied as shown in Fig. 6. The change in the real part of the effective index implies a huge shift in the resonance wavelength and is estimated by ${\rm{\Delta }}{\lambda }_{res}=\frac{{\rm{\Delta }}{n}_{{\rm{e}}ff}L}{m}=\frac{{\rm{\Delta }}{n}_{eff}L}{L/{\lambda }_{eff}}=\frac{{\rm{\Delta }}{n}_{eff}}{{n}_{eff}}\,{\lambda }_{o}\approx 100\,nm$.

## Design Optimization

Once the minimum losses inside the ring have determined, as described hereafter, the required coupling coefficient can be estimated for optimum performance. For the chosen radius of 1 µm, our initial estimate for the round trip transmission coefficient (α) is ~0.77, the device is not allowed to operate in the shaded area of Fig. 2. From Fig. 2, we can deduce there is a trade-off between the extinction ration and the minimum possible insertion loss of the modulator that is defined as the reciprocal of the transmission when α = 0. As the coupling strength increases, the insertion loss increases. The transmission coefficient should be chosen to achieve a good compromise between the insertion loss and the extinction ratio.

**Figure 7:**
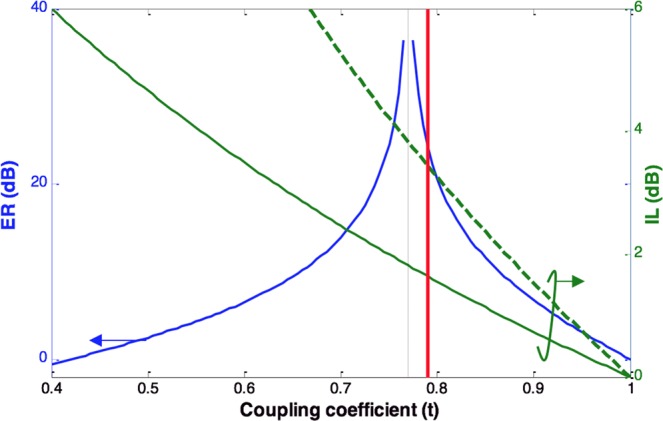
Extinction ratio and Insertion loss vs coupling coefficient (t); The insertion loss without index modulation, (dotted line), with index modulation (solid line).

To find the optimum coupling strength for α around 0.77, we plotted the insertion loss and the extinction ratio assuming a loss of 2 dB/µm for case with external voltage applied. As evident from Fig. 7, the maximum extinction ratio is at the critical coupling (represented by the dotted line) when t = α. The figure depicts the variation of the insertion loss in two cases: a) the hypothetical case when the ring is only loss modulated (dashed line), b) the waveguide ring resonator experiences changes in both the imaginary and real part of the effective index (solid line). The fact that our proposed ring modulator utilizes index and loss modulation improves the insertion loss and keeps it below 2 dB for *t* values above and around α. In the proposed modulation scheme, the insertion loss is also less sensitive to the coupling coefficient variations caused by temperature and fabrication process.

### Coupling junction design

A point coupler is utilized to couple the light from the pure silicon waveguide to the hybrid plasmonic waveguide. As discussed earlier, the coupling strength between the two waveguides is inherently weak due the phase mismatch. One possible way of increasing the coupling is to decrease the gap (g) between the ring and the bus waveguide. However, as far as the fabrication process is considered, the minimum feature size is 100 nm in 193 nm deep UV photolithography^28^. So, a gap size of 100 nm has been chosen.

Since the coupler is not symmetric, two coupling coefficients t_1_, t_2_ are defined. To account for the losses of the coupler, we also define α_1_ and α_2_. The subscript “1” annotates the coefficients when the excitation source is at the silicon bus waveguide (port 1) while subscript “2” annotates the case when the light is excited from the HP waveguide ring waveguide (port 2).

There are two main sources of losses in the coupler; (1) higher order modes excited inside the access waveguide at the coupling junction due the phase mismatch. Then the power in these modes turns into radiation since they are not supported by the access waveguide, (2) The parasitic polarization rotation that occurs at the junction. When port 2 is excited by a TM HPW, the output power at port 3 can expanded into the 2 fundamental TE and TM modes with ratios 0.13 and 0.82, respectively. This simply means that every roundtrip some power is lost to the TE mode.

**Figure 8:**
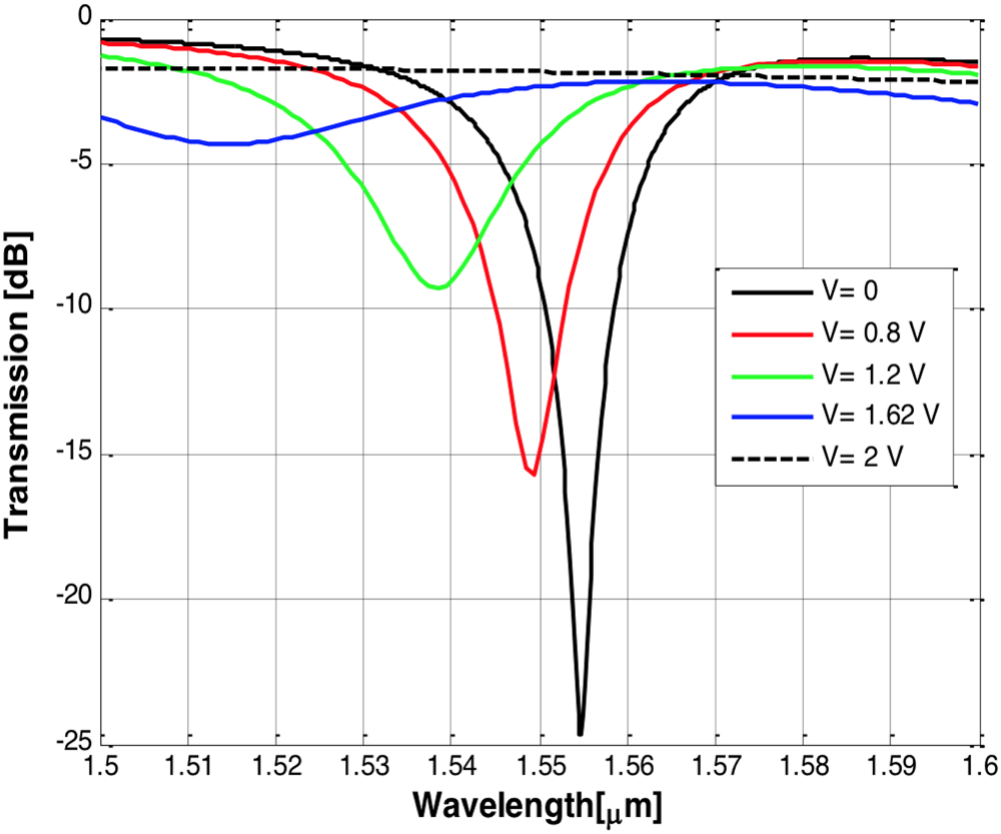
Voltage modulation of the ITO ring modulator.

We made minor modifications to the analytical model of the ring resonator to take into consideration the asymmetry and losses of the coupler. The power transfer characteristics can be deduced using the coupled mode theory^29^. The on-resonance transmission is expressed as

6 $$T={a}_{1}^{2}\frac{{t}_{1}^{2}+{a}^{2}-2a{t}_{1}}{1+{a}^{2}{t}_{2}^{2}-2a{t}_{2}}={a}_{1}^{2}\frac{{({t}_{1}-a\zeta )}^{2}}{{(1-a{t}_{2})}^{2}}$$

where α_1_ represents the losses of the coupler when the input is at port 1, a represents the total round trip losses and ${\rm{\zeta }}$ represents the asymmetry coefficient. We choose the width of the silicon strip access waveguide that achieves the highest coupling efficiency and minimizes the losses. Using finite difference time domain simulations, we estimated the coupling coefficient (t) for different access waveguide widths (W_strip_) for a HPW of width 200 nm and a gap size of 100 nm as depicted in Fig. 8. The radius is chosen to be 1 µm as we discussed earlier. For the aforementioned HP waveguide width and radius, the transmission coefficient of the ring is optimized to achieve the critical coupling condition which is satisfied once t_1_ and α are equal. Hence, in our design of the coupling junction, we tune the design parameters so that the values of t_1_ and α_2_ satisfy the critical coupling coefficient. As such an optimum width for the access waveguide of 350 nm is utilized to achieve the critical coupling condition.

For this width, an ER of 24 dB and insertion loss (IL) of 1.64 dB is achieved as indicated by the red line in Fig. 7.

## Discussions and Conclusion

The transmission spectra of the ring modulator in both the on and off states is studied using 3D finite difference time domain simulations. The output of the TM excited ring exhibits a resonance notch in its spectrum of depth of 25 dB at the wavelength of 1555 nm when no external voltage is applied as shown in Fig. 8.

To estimate the speed of the device, three factors play significant roles. First, the speed of accumulation layer formation which was shown to exceed the THz speed as discussed earlier. Second, the loaded quality factor is $Q=\frac{{{\rm{\lambda }}}_{{\rm{o}}}}{{\rm{\Delta }}{\lambda }_{FWHM}}=\frac{1555}{15.97}=97.4$ which allows a modulation bandwidth of ${\rm{\Delta }}f=2THz$. Third, the capacitance is estimated, assuming parallel plate capacitor, by $C/A=\frac{{{\epsilon }}_{0}{{\epsilon }}_{r}}{{t}_{ox}}=0.93\,fF/\mu {m}^{2}$. So, the total capacitance of the device C = 1.2 fF. Assuming a 100 Ω resistance for the interconnects and the device, the capacitive RC cut-off frequency is 1.3 THz. Since the cut-off frequency is lower than the modulation bandwidth allowed by the quality factor of the resonator, the speed of the device is limited by the capacitive loading effect rather than the resonator line-width. Of course the practical bandwidth will depend on the fabrication technology and quality of the contacts. However, the high modulation speed limit reported here allows for fast modulation development assuming high quality fabrication technology.

When the voltage is applied, the resonance notch is blue-shifted, broadened and exhibits a lower extinction ratio as shown in Fig. 8. The electric field intensity at the original resonance wavelength observed at the silicon waveguide output port increases as the applied voltage increases. The power gradually exits the cavity to the bus waveguide so that the modulator is switched from OFF to ON state.

For a voltage of 1.62 V, the loss of the waveguide loop is about 2 dB/µm and the effective index decreases about 0.075. Hence, the 3D FDTD simulations show the insertion loss is 1.6 dB and the modulation depth reaches 22.8 dB. The energy per bit achieved is 3.25 fJ/bit. These performance figures agree very well with the values obtained using our earlier analysis. Further increase of the voltage doesn’t improve the performance significantly. This performance is superior to non-resonant conducting oxide modulators and to plasmonic ring modulators operating under the same power budget. The performance is also superior to the plasmonic ring resonator based modulator which is fabricated using comparable ring dimensions made using metal slot configuration with higher intersic loss and lower ER due to the low quality factor of the ring^30^.

To conclude, a hybrid plasmonic waveguide ring resonator coupled to a standard SOI photonic waveguide was proposed and analyzed. A stack of indium-tin-oxide, SiO_2_ and Noble metal is placed atop the ring’s silicon waveguide. While similar structure was experimentally demonstrated in an earlier pioneering work as electro-absorption modulator, we are the first to demonstrate its use in a ring configuration to achieve a high performance modulator. Challenges in the design of the coupling junction have been thoroughly addressed.

## Methods

### Optical properties of carrier-injected ITO

The permittivity of ITO can be described by the Drude model^18,22,23^ as:

$${{\epsilon }}_{r}={{\epsilon }}_{\infty }-\frac{{\omega }_{p}^{2}}{{\omega }^{2}-i\gamma \omega }$$

Where ${{\epsilon }}_{\infty }\,$= 3.9 is the high frequency dielectric constant, *γ* = 2.89 × 10^14^ rad/sec is the electron scattering rate, ${\omega }_{p}$ is the plasma frequency and $\omega$ is the frequency of light. The plasma frequency depends on the carrier concentration *N*_*c*_ as follows:

$${\omega }_{p}^{2}=\frac{{N}_{c}{e}^{2}}{{{\epsilon }}_{o}{m}^{\ast }}$$

Where m^*^ = 0.35 *m*_*o*_ and *m*_*o*_ is the electron effective mass, *e* is the electron charge and ${{\epsilon }}_{o}$ is the permittivity of free space. Accordingly, the permittivity for the On and Off cases depends on the carrier concentration as described in Table 1.

## Supplementary information

Supplementary material

## Data Availability

All data needed to evaluate the conclusions in the paper are present in the paper and/or the Supplementary Materials. Additional data related to this paper may be requested from the authors.
