# Supplementary material for: On Chip Optical Modulator using Epsilon-Near-Zero Hybrid Plasmonic Platform
Source: Sci Rep. 2019 Apr 30;9:6669. doi: 10.1038/s41598-019-42675-z (PMC6491601; doi:10.1038/s41598-019-42675-z)
Supplement: Supplementary file 1 — Supplementary material [file 41598_2019_42675_MOESM1_ESM.docx]

On Chip Optical Modulator using Epsilon-Near-Zero Hybrid Plasmonic Platform

Mohamed A. Swillam^1*^, Aya O. Zaki,^1,2^ Khaled Kirah,^2^ and, Lamees A. Shahada,^3^

^1^Department of Physics, School of Science and Engineering, The American University in Cairo, New Cairo, 11835, Egypt.

^2^Engineering Physics Dept., Faculty of Engineering, Ain Shams University, Abassia, Cairo, 11517

^3^Department of Chemistry and Earth Sciences, College of Arts and Science, Qatar University, P.O. Box 2713, Doha

*m.swillam@aucegypt.edu

**Supplementary Material**

Since the coupler is not symmetric, we defined two coupling coefficients $t_{1},t_{2}$ . The subscript “1” annotates the coupling coefficient when the excitation source is at the silicon bus waveguide (port 1) while subscript “2” annotates the case when the light is excited from the HPW ring waveguide (port 2).

To account for the losses of the coupler, we also define $\alpha_{1}$and $\alpha_{2}$. There are two main sources of losses in the coupler:

1. Higher order modes excited are inside the access waveguide at the coupling junction due the phase mismatch. Then the power in these modes cannot propagate along it and turns into radiation since they are not supported by the access waveguide.
2. The parasitic polarization rotation that occurs at the junction. When port 2 is excited by a TM HPW, the output power at port 3 can expanded into the 2 fundamental TE and TM modes with ratios 0.13 and 0.82, respectively.


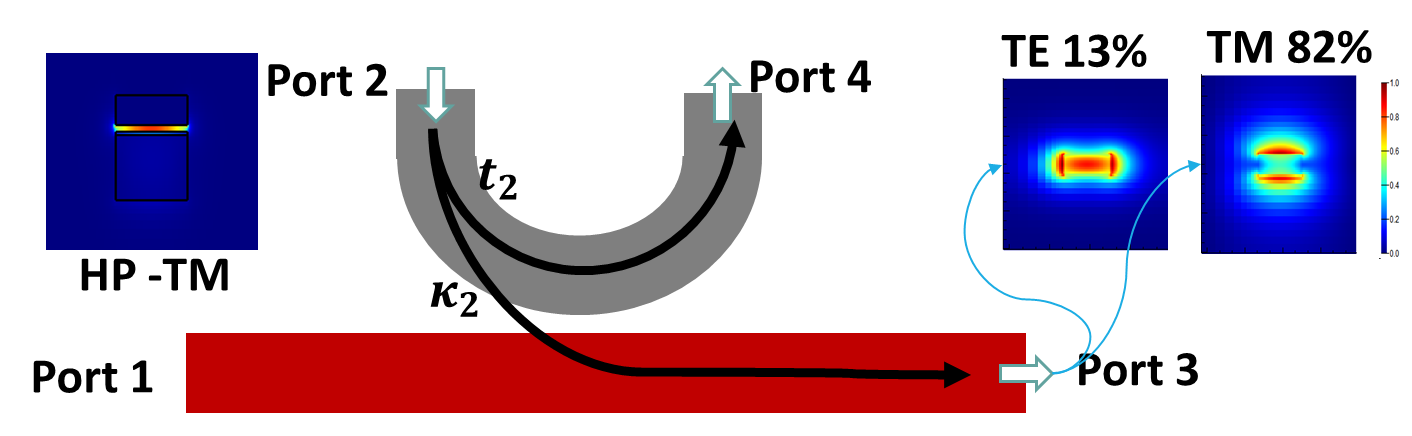


**Figure S1. Schematics of the coupling junction**

This means that every roundtrip some power is lost to the TE mode. This power creates a peak at the resonance frequency in the transmission spectrum at the output of the access waveguide as shown in Figure ‎S2 .

We made small changes to the analytical model of the ring resonator to take into consideration the asymmetry and losses of the coupler. The feedback block diagram below illustrates our model. The power transfer characteristics can be deduced as follows:

| $E3=t_{1}E1 +i\kappa_{2}E2$ $E4=i\kappa_{1}E1+ t_{2}E2$ $E2=E4 . ae^{i\theta}, \theta=\beta L$ $\therefore E4 \left( 1-t_{2}ae^{i\theta} \right)=i\kappa_{1}E1$  $\frac{E3}{E1}=t_{1}+i\kappa_{2}.ae^{i\theta}.\frac{i\kappa_{1}}{\left( 1-t_{2}ae^{i\theta} \right)}=\frac{t_{1}\left( 1-t_{2}ae^{i\theta} \right)-\kappa_{1}\kappa_{2}ae^{i\theta}}{1-t_{2}ae^{i\theta}}=\frac{t_{1}-ae^{i\theta}\left( t_{1}t_{2}+\kappa_{1}\kappa_{2} \right)}{1-t_{2}ae^{i\theta}}$ | | (S – 1)  (S – 2)  (S – 3)  (S – 4) |
| --- | --- | --- |
| ${T=\left\vert\frac{E3}{a_{1}E1} \right\vert}^{2}=\frac{t_{1}^{2}+a^{2}\zeta^{2}-2a\zeta t_{1}\cos\left( \theta\right)}{1+a^{2}t_{2}^{2}-2 at_{2} cos(\theta)}$ | (S – 5) | |


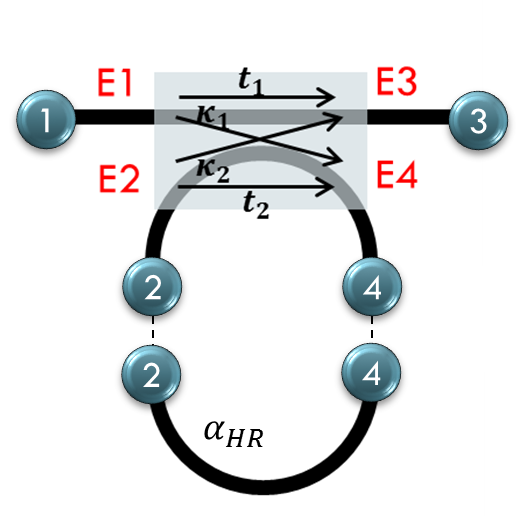


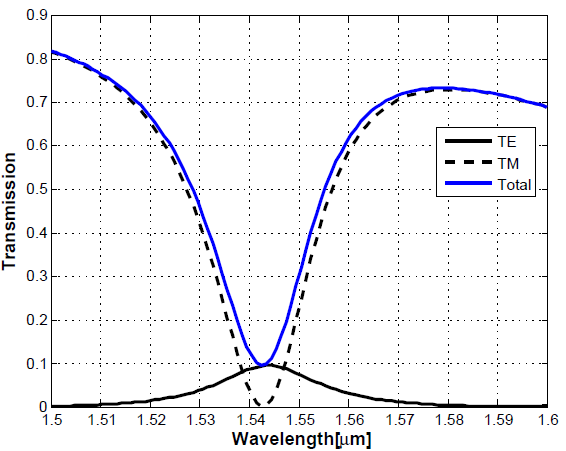


**Figure S2. Transmission spectra at the output of the access waveguide when the resonator is excited by a TM mode; (solid line) Spectrum of the TE polarized transmission, (dotted line) Spectrum of the TM polarized transmission.**

| At resonance, $\theta=2m\pi$  $T=a_{1}^{2}\frac{t_{1}^{2}+a^{2}-2at_{1}}{1+a^{2}t_{2}^{2}-2 at_{2}}= a_{1}^{2}\frac{\left( t_{1}-a\zeta\right)^{2}}{\left( 1-at_{2} \right)^{2}}$ | (S – 6) |
| --- | --- |

The round-trip phase shift

$$\phi=\frac{2\pi}{\lambda}n_{eff}L$$

$\lambda$ is the incident wavelength, $n_{eff}$ is the ring effective index, $L$ is the ring circumference.
